# Tools for measuring curriculum integration in health professions’ education: a systematic review

**DOI:** 10.1186/s12909-024-05618-5

**Published:** 2024-06-06

**Authors:** Soumaya Allouch, Raja Mahamade Ali, Noor Al-Wattary, Michail Nomikos, Marwan F. Abu-Hijleh

**Affiliations:** 1 Basic Medical Sciences Department, College of Medicine, QU Health, Qatar University, PO Box 2713, Doha, Qatar; 2 College of Education, Qatar University, PO Box 2713, Doha, Qatar

**Keywords:** Integrated curriculum, Instrument, Questionnaire, Health professions, Medical education

## Abstract

**Background:**

Curriculum integration is an important educational concept widely implemented by various educational institutions, particularly within the healthcare field. Its significance lies in enhancing the preparation of future healthcare professionals. The assessment of these integrated curricula is imperative to guarantee their effectiveness. Consequently, the aim of this systematic review is to delve into existing literature, with the goal of identifying instruments designed to assess the extent of curriculum integration in health professions’ education.

**Methods:**

A comprehensive search was conducted to identify peer-reviewed papers and grey literature describing the development, validation, or use of instruments measuring the degree of integration in a curriculum. Eight databases were searched: PubMed, Scopus, Google Scholar, CINAHL Ultimate, Web of Science, Cochrane, ProQuest Central and EMBASE. Grey literature was also included. Titles, abstracts, and full text screening was conducted. Data extraction was done using a data extraction tool developed by our research team.

**Results:**

The search resulted in the identification of 2094 references. After the removal of duplicates and title and abstract screening, 16 articles were deemed suitable for inclusion in this systematic review. Twenty-two instruments were extracted from these articles. The identified instruments assessed either integration attributes, perceptions about the integrated curriculum characteristics, process and outcomes, or curriculum integration level. Two of the instruments were focused on assessing horizontal integration (*Basic Science Curriculum Assessment Instrument* and *the integration characteristic tool).* In addition, one instrument was developed to assess integration within a single session only, while other instruments assessed curriculum integration level. Two of the integration instruments (*The Session Integration Tool* and *Integration Ladder Questionnaire)* provided scales for calculating integration levels. Validation of the integration assessment instruments was infrequent, with only 9 of 22 instruments validated for their psychometric properties.

**Conclusion:**

Our findings reveal the existence of diverse instruments designed to assess the extent of curriculum integration within health professions’ curricula. The majority of identified instruments were focused on participants’ perceptions towards the attributes of the integrated curriculum, and a significant number of these tools lacked validation.

**Supplementary Information:**

The online version contains supplementary material available at 10.1186/s12909-024-05618-5.

## Background

Curriculum integration is a concept which has been frequently discussed in educational literature for the past 3 decades [1]. Introduction of this concept was accompanied by attempts to reform medical curriculum from the traditional discipline-based curriculum to an integrated one; with the first documented attempt in McMaster University, Canada [1]. The primary reason for this shift is the recognition that conventional discipline-based curricula fall short in equipping medical students with the necessary skills to effectively apply their acquired knowledge in clinical practice [2]. While the term ‘integration’ is commonly used throughout the literature, a clear consensus on its definition within medical curriculum remains elusive. For instance, a recent systematic review by Matinho et al. (2022) on integrated learning in health professions’ education found that only 37% of relevant studies provided a clear definition of integration [3]. These definitions belonged to one of three main categories which described integrated learning as: (1) the extent to which educators from different disciplines co-present knowledge, beliefs or skills to students; (2) the organizational approach that informs how curricular elements are structured and arranged; and (3) the description of the cognitive or metacognitive processes occurring within the learners’ mind. The first category is well aligned with Harden’s definition, which states that integration is *‘the organisation of teaching matter to interrelate or unify subjects frequently taught in separate academic courses or department’* [4]. The second category identified in the systematic review aligns with Brauer & Ferguson’s perspective on integrated curriculum, which views it as ‘*a fully synchronous, trans-disciplinary delivery of information between the foundational sciences and the applied sciences throughout all years of a medical school curriculum’* [1].

Matinho et al.’s (2022) systematic review also highlights the practical implementation of this definition through vertical, horizontal, and spiral integration [3]. Horizontal integration refers to the integration across different subject areas within a finite period of time, while vertical integration refers to the integration between basic and clinical disciplines across time [2, 5]. In vertical integration the amount of time spent on classroom education tends to decrease gradually as more clinical practice experience is introduced [5]. Integration in its most ideal form represents a combination of both horizontal and vertical, uniting integration across time and across disciplines, which has often been termed as ‘spiral integration’ [1, 6]. These levels of integration in medical curricula are described as a continuum, or spectrum. This ranges from traditional curriculum design, where contents are taught as separate disciplines, to a highly innovative integrated approach where disciplinary boundaries are abandoned [7].

In the integrated curricula, teaching revolves around themes upon which the content of individual units is developed [8]. This approach encourages students to see the link between different subjects and helps them understand applications of this knowledge in practice. An important model of integration, which was developed for curriculum planning and review, is the Student-Centered, Problem-Based, Integrated, Community-Based, Elective, Systematic (SPICES) model [4]. This model describes a set of six educational strategies arranged in a continuum across two extremes, ranging from the least desirable traditional curriculum to the most desirable innovative curriculum. In the new integrated curriculum students are the focus of the learning experience, and they are given liberty to determine their learning objectives, learning resources, and sequence of their learning content under instructors’ guidance [4]. Problem-based learning (PBL) is the learning tool of choice in this model. PBL is a small group learning approach (8–10 students), in which students are provided with a problem they need to solve through conducting research, reviewing relevant resources, and integrating theory within practice [9]. The process is facilitated by a tutor who supports students and provides a thorough debriefing at the end of the PBL session [9]. Another important model of integration is Harden’s integration ladder, which consists of eleven steps describing the integration degree of a curriculum on a continuum ranging from isolation (no integration) to multidisciplinary (fully integrated curriculum) [10]. A more detailed model is Fogarty’s integration model which classifies integration levels according to where integration is adopted into three broad categories; within single disciplines, across several disciplines and within and across learners [11]. This model generated ten integration levels ranging from fragmented curricula, in which integration is absent, to networked curricula, in which integration of knowledge occurs within and across the learners’ mind as they direct the integration process both internally and externally (determining needed resource, expert matter experts…etc.) [11, 12]. All these models can be used to guide the planning, development, or evaluation of integrated curricula.

Several studies report that integrated medical curricula demonstrate greater effectiveness compared to conventional curricula [13–15]. Students from integrated medical programs were shown to perform better in examinations of medicine, pediatrics, obstetrics and gynecology subjects when compared to students from traditional curricula [14, 16]. Additionally, it was reported that graduates from integrated curricula tend to make definitive career choices earlier, are more likely to be accepted at residency positions faster and are more confident in their readiness for practice [13]. Similarly, PBL was found to be superior to traditional learning methods in enhancing students’ social and communication skills, as well as advancing their problem solving and self-learning skills [17]. It was also reported that students from integrated curricula and those exposed to PBL have superior diagnostic skills compared to students from traditional curricula [15]. Learning theories suggest that the integrated approach of teaching and learning enhances students’ learning, engages adult learners’ interest in meaningful learning, and improves retention of knowledge [18–20]. Integrated curricula are designed to encourage students to establish connections between various subjects, thus enabling them to recognize how their knowledge can be applied to real-world patient cases [8]. In addition, integrated curricula provide students with opportunity to engage in self-directed learning and develop clinical reasoning skills. This also allows students to express their personal identities and individual qualities while learning, and as a result helps them in developing their individual attributes as future healthcare providers [8].

Despite the challenges of defining integration, there are domains and dimensions to the construct that provide guidance and boundaries for defining what constitutes integration [21]. The general assumption is that integration should promote retention of knowledge and acquisition of skills through repetitive and progressive development of concepts and their applications [1]. Different educational institutions, particularly in the health profession field, adopted integration in their curriculum to prepare their students for practice [3]. However, for these curricula to be effective, it is a paramount to evaluate them and assess the degree and extent of curriculum integration following its implementation using appropriate tools. These tools will help educators and curriculum developers in identifying gaps in their curriculum design pertaining to integration and provide suitable solutions. Therefore, the aim of this systematic review is to explore the current literature to identify tools, instruments, or surveys, which have been developed to assess the degree of curriculum integration in health professions education.

### The research question

What tools, instruments, or surveys are available for measuring the degree of curriculum integration in health professions education?

## Methodology

This systematic review was conducted and reported in accordance with the Preferred Reporting Items for Systematic Reviews and Meta-Analyses (PRISMA) checklist for systematic reviews 2020 [22].

### Search strategy

A comprehensive search was conducted in 8 large databases: PubMed, Scopus, google scholar, CINAHL Ultimate, Web of science, Cochrane, ProQuest central and EMBASE. The aim was to identify peer-reviewed papers and grey literature describing the development, validation, or use of instruments measuring the degree of integration in health professions’ curriculum. To identify relevant articles, the search was conducted in these eight databases using different combinations of the keywords listed below:

- Tool, instrument, survey, questionnaire, scale, measure.
- Curriculum delivery, curriculum evaluation, curriculum assessment.
- Integrated curriculum, vertical integration, horizontal integration, spiral integration, basic sciences integration, clinical sciences integration, clinical and basic sciences’ integration.
- Medical education, medical school, medical college, health professions education.
- Problem based learning, PBL, student centered curriculum.

The full search strategy for the PubMed database can be found in Additional file 1.

### Inclusion and exclusion criteria

Peer-reviewed articles or grey literature published in English up until October 2, 2023 were included in the search. Grey literature (e.g., conference proceedings, thesis, dissertation etc.), relevant to our study identified through the search was also included. Evidence, including questionnaires or instruments assessing the degree of curriculum integration (as the construct of the instrument or one of the main constructs if the instrument consists of multiple constructs/domains) in health professions’ education, was included in this study. Studies were eligible for inclusion if they incorporated the questionnaire or questions assessing the degree of integration within the article. Studies describing such tools and their questions (whether validated or not) were also included. Articles not fulfilling our inclusion criteria were excluded from this systematic review.

In the first phase, one researcher screened titles yielded from the database search, aiming to identify relevant articles for inclusion in the study. In the second phase, three researchers independently screened abstracts and keywords of articles identified in step one. In the final step, three researchers thoroughly reviewed the full texts of papers that successfully passed the previous screening. Only papers that presented or described an instrument, tool or survey assessing curriculum integration were considered eligible for inclusion. Additionally, we conducted a manual search to identify any additional relevant papers which might have been missed. All eligible articles, once identified, underwent screening for inclusion. Any conflict or disagreement between reviewers at any stage of the review was resolved through discussion or involvement of a third researcher who was involved in the study since early stages and is aware of the inclusion and exclusion criteria. Reasons for exclusion of citations were documented and are reported in the results section of this systematic review. The search process is reported in detail per the PRISMA flow diagram [22]. *Characteristics of included papers*:

This study only included observational studies describing the development and/or use of an instrument evaluating curriculum integration (e.g. cross-sectional studies, longitudinal studies, cohort studies etc.). Following the search of the selected databases, all identified citations were uploaded to Mendeley citation management software 2.95/2023 (©2023 Mendeley Ltd., Elsevier), and duplicates were removed [23]. Articles were then transferred to Rayyan, which is a web application that facilitates the process of abstract and full text screening of articles included in a review by different team members [24]. The tool also identifies and highlights disagreements between different researchers, and documents decisions made regarding articles screened.

### Data collection process

We extracted data from studies that met the inclusion criteria using a data extraction tool developed by our research team. The extracted data included information about the papers, such as authors’ names and publication years, and data on the instruments, including their names, objectives, main domains measured, and other details such as number of items, scale, and scoring system. If the instrument was utilized within a sample, details about this sample were extracted from the relevant paper and are reported in this review. Psychometric properties were also extracted if they were reported in the retrieved articles.

### Quality assessment

The quality of the papers included in this review were assessed using the Risk of Bias Utilized for Survey Tool (ROBUST) [25]. The tool consists of 8 criteria which measures sample frames, participant recruitment, exclusion rate, sample size, measurement validity, setting and data management. If the study met the criteria a score of 1 corresponding to “yes” is given while a score of “0” corresponding to “No” are assigned for studies which fails to meet the criteria. For the purpose of this study, we modified the tool by removing the “exclusion rate” item as this element is not reported in our studies. Additionally, the criteria for sample characteristics have been modified as in our study (response “yes” represents reporting the college and academic year/s). The total score for quality ranges between 0 and 7; such that 0 represent the lowest level of confidence in the results and 7 the highest confidence level.

## Results

**Fig. 1:**
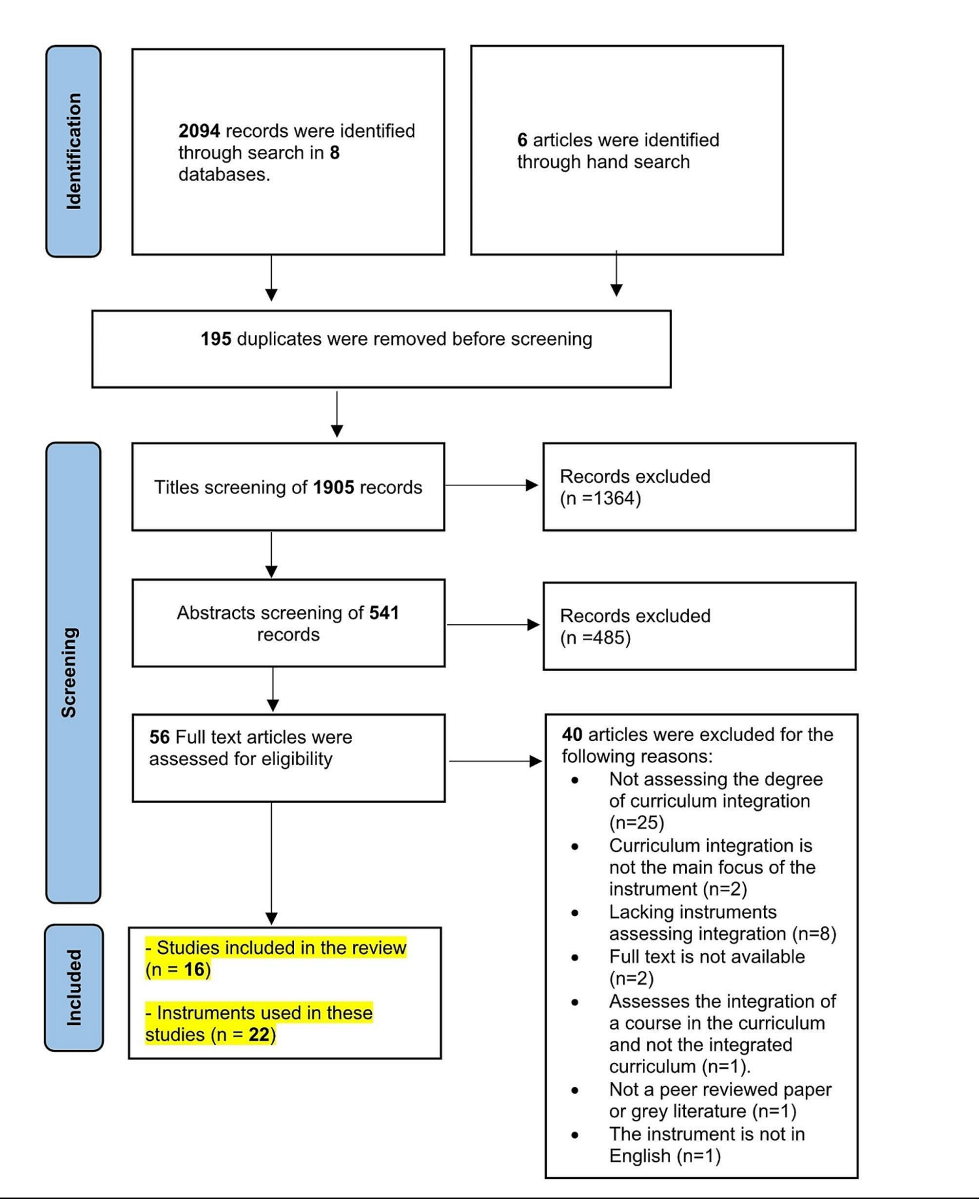
PRISMA flow diagram summarizing the search strategy followed to identify instruments assessing the degree of curriculum integration in health professions’ education

The search resulted in the identification of 2094 references. Six additional articles were identified through manual search. After eliminating duplicates, a total of 1905 references were retained and underwent title and abstract screening. After full text screening, 16 articles fulfilled this study’s inclusion criteria and were deemed suitable for inclusion in this systematic review. Details of the search strategy are presented in Fig. 1. Twenty-two instruments assessing curriculum integration in health professions’ education were extracted from these articles.

The papers included in this review were published between 1980 and 2023, with the majority published after 2010 (*n* = 12). All identified instruments were questionnaires developed to assess participants’ perception of curriculum integration or integration level, except for one instrument, which was administered as an assessment rubric of integration competency during an Objective Structured Clinical Examination (OSCE) [26]. Additionally, all instruments identified focused on assessing integration on a curricular level except for one instrument; the Session Integration Tool (SIT), which was developed to measure the level of integration between different disciplines in a single session [27].

Most of the included papers were centered on capturing students’ perceptions and experiences with curriculum integration (*n* = 11) [26, 28–37]. In contrast, some were specifically designed to investigate faculty members’ opinions and evaluations of the curricula and its level of integration [27, 37–39]. Notably, in seven studies, integration-assessing questionnaires were distributed to both students and faculty members to benefit from the perceptions and unique experiences of each group [26, 29–31, 33, 35, 36]. Only two studies focused on evaluations of academic leaders and expert evaluators [40, 41].

Half of the integration assessment questionnaires identified in this review were developed to evaluate integration in medical curricula (*n* = 11), and targeted either students, faculty members or integrated course’s developers or directors at medical schools. The remaining questionnaires (*n* = 11) were developed to assess integration in other health professions’ curricula namely physical therapy, nursing, pharmacy, dentistry, radiography and health sciences. More details of integration instruments and the samples in which they were used is provided in Tables 1 and 2.

**Table 1:** Instruments’ domains

Study title, (Author, year of publication)	Instrument name	Purpose of the instrument	Domains
Assessing clinical competency in the health sciences (Panzarella, K. 2003).	Integrated Standardized Patient Examination (ISPE) *A component of the OSCE assessment rubric.*	To assess the Students’ clinical competence including their ability to integrate basic science knowledge with clinical practice. (assessed through students’ responses to Standardized patients’ questions).	The rubric consists of 4 sections: ▪ History ▪ Integration ▪ Physical examination ▪ Overall evaluation
	Student feedback form	Captures students’ opinion of their own performance during the encounter with the SP.	Perception of their performance (integration competency). Readiness for the encounter. Perception of the patient case.
	Evaluator feedback form	Assesses students’ performance during their interaction with the SP (i.e. integrating the knowledge and skills).	Perception of students’ performance (integration competency) Perception of the patient case. Perceptions of integration.
Student’s perception regarding an integrated curriculum at a public sector medical college (Lajber et al., 2020)	NR	To assess medical students’ perception on integrated curriculum’s content and delivery.	Four main domains: ▪ Content coherence ▪ Subjects’ time management ▪ Teaching and learning methods ▪ The assessment methods.
Nursing faculty perceptions of an integrated curriculum and implementation of the curriculum (Strandell, CH., 1980)	Integrated curriculum in nursing inventory	To collect nursing faculty perceptions of an integrated curriculum in nursing.	Three domains assessing perceptions about: **Section A**: the meaning of “integrated curriculum”. **Section B**: the current integrated curriculum (i.e. characteristics of the curriculum). **Section C**: how the current “integrated curriculum” should be.
	Integrated curriculum implementation inventory	Measures the degree of implementation of an integrated curriculum in the school studied.	One domain
Delivering endocrinology and reproduction in an integrated modular curriculum (Ghayur et al., 2012)	Student feedback	Assesses the acceptability of the module by the Students.	One domain
	Faculty feedback	Obtain faculty members feedback on the integrated module	One domain
Developing tool and measuring integration characteristics of basic science curriculum to improve curriculum integration (Maharjan, 2018)	The integration characteristic tool	It measures the perception of students /faculty members on the integration characteristics of basic science curriculum.	NR
Survey of pharmacy schools’ approaches and attitudes toward curricular integration (Poirier T. et al., 2016)	Integration survey	To identify how integration is addressed in pharmacy schools in the US.	NR
Integration of basic and clinical science courses in US PharmD programs (Islam M. et al., 2016)	Faculty perception of curricular integration survey	To investigate the integration of basic and clinical science courses in US doctor of pharmacy (PharmD) programs.	**Section 1**: perceptions of curricular integration. **Section 2**: the current status of integrated course provision in pharmacy colleges, the design and implementation of integrated courses. **Section 3**: barriers and challenges to the implementation of integrated courses. **Section 4**: demographics of faculty members
Evaluation of the first curriculum year of the new integrated and interactive curriculum at the university of medicine and pharmacy at Ho Chi Minh city (ump), Vietnam (Le B. K., 2018)	Student online survey questionnaire	Assessing the new curriculum processes, Outcomes, impacts and implementation.	▪ Preparation of lessons before class. ▪ Interaction between faculty and students. ▪ Integration in teaching and student assessment.
	Faculty Online Survey Questionnaire		▪ Preparation of lessons before class. ▪ Interaction between faculty and students. ▪ Integration in teaching and student assessment. ▪ Faculty development activities.
Evaluating dental students’ perspectives on the concurrent teaching of didactic and case-based courses (Parikh et al., 2022)	Students’ survey	To evaluate student perception of integrating biomedical and clinical Sciences.	NR
An integrated learning curriculum for radiography in South Africa (Engel-Hills P. C., 2005)	Questionnaire on Radiography education and Training in south Africa.	This questionnaire explores the opinions of stakeholders on the status of radiography education.	7 domains. Section G is specific for curriculum integration. **Section G**: program design (focuses on assessing curriculum integration.)
Evaluating the construct validity of basic science curriculum assessment instrument for critical thinking: a case-study (Chen C. et al., 2018)	Basic Science Curriculum Assessment Instrument	Assesses students’ perceptions of basic science characteristics and impact on integration of knowledge	NR
Evaluation of Integrated Teaching Method for Phase I MBBS, Using Kirkpatrick’s Evaluation Method (Dohnde et al., 2020)	Questionnaire for students’ feedback	These tools explore opinions about the integrated teaching method and its usefulness.	▪ Perception about teaching & learning by integrated teaching. ▪ Perception about organization of integrated teaching program.
	Questionnaire for faculty feedback		NR
Simulated patient videos to supplement integrated teaching in competency based undergraduate medical curriculum (Nayak K. R. et al., 2023)	NR	Collect students and faculty feedback on integrated teaching modules	▪ Timetable and time management ▪ Content coherence ▪ Teaching learning ▪ Resources for learning ▪ Evaluating student satisfaction
An integrated curriculum: evolution, evaluation, and future direction (Howard K. et al., 2009)	NR	To evaluate a dental school curriculum to determine the extent of vertical and horizontal integration originally intended.	NR
A tool for evaluating session‑level integration in medical education (Heck A., Chase A., 2021)	The session Integration Tool (SIT) according to 3 criteria for the integrated curriculum in medical education guide#96.	Assesses session level integration	▪ *Development* (planning prior to course delivery) ▪ *Delivery* (presentation of material to the students) ▪ *Outcomes* (what can be demonstrated by the end of the session)
Level of integration in current undergraduate curricula of two private-sector medical colleges in Karachi (Baig et al., 2022)	Integration ladder questionnaire	The tool was developed to assess the current level of curriculum integration in medical institutions.	NR

**Table 2:** Instruments’ details and psychometric properties

Study title (Author, publication year)	Sample	Curriculum details	Details about the instrument	Psychometric properties
Assessing clinical competency in the health sciences (Panzarella, K. 2003).	Second year doctor of physical therapy students **Curriculum style**: Integrated curriculum **Learning method**: NR	Integrated curriculum	**Number of questions**: 4 (the Simulated Patients (SPs) asks specific questions assessing knowledge integration). **Scale**: 4-point grading scale in which (0: detrimental, 1: below expectations, 2: meets expectations, 3: exceeds expectations)	**Integration section**: ***Inter-rater reliability*** (Cohen’s kappa): ▪ Expert-investigator: 60.3 (0.18) moderate ▪ Expert-student: 53.0 (0.12) low ▪ Investigator-student: 53.0 (0.08) low ***Internal consistency***: Ranged between **0.47 and 0.69** ***Content validity***: Cases and SP questions assessed students’ ability to integrate their knowledge. ***Construct validity***: Determined through feedback about the ISPE and its ability to measure the intended construct.
	Second year doctor of physical therapy students (Buffalo university, US).		**Number of questions**: 9; 4 close ended questions and 5 open ended questions. **Scale**: 4-point Likert scale: (1: Extremely well, 2: Well, 3: Fairly well, 4: Poorly) Definitions of integration and competency were provided.	NR
	Expert evaluators (Physical therapy faculty members and clinicians).			NR
Student’s perception regarding an integrated curriculum at a public sector medical college (Lajber et al., 2020)	Second year MBBS students at Bacha khan medical college, Pakistan (*N* = 50)	Integrated curriculum	**Number of questions**: 11 **Scale**: 3-points Likert (1: Agree, 2: Disagree, 3: Neutral) Brief introduction about integrated teaching was given.	NR
Nursing faculty perceptions of an integrated curriculum and implementation of the curriculum (Strandell, CH., 1980)	Baccalaureate nursing faculty from different programs in the US (**n =** 187)	Integrated curriculum	**Number of questions**: 138 (each section has 46 questions). **Scale**: 4-point Likert scale (1: Strongly disagree, 2: Disagree, 3: Agree, 4: Strongly agree)	**Reliability** (*N* = 186): Section A: Reliability coefficient (r) = 0.929* Section B: *r* = 0.950* Section C: *r* = 0.951* **Content validity**: ▪ Literature on curriculum integration was the source for developing the questionnaire items. ▪ 20 self-study reports from nursing schools with integrated curriculum were reviewed. ▪ Nursing faculty opinions about the instrument items and their suggestions were collected. ▪ Authoritative opinions of experts.
	Researchers and two raters who reviewed the included school curricula.		**Number of questions**: 25. **Scale**: 3-points Likert scale (3: High implementation, 2: Moderate implementation, 1: Negligible implementation)	**Content validity**: ▪ Sources for developing the questionnaire included literature on nursing curriculum, the national League for nursing Reports, the criteria for the appraisal of baccalaureate nursing programs and the opinions of nursing educators. ▪ Authoritative opinions of curriculum and nursing experts.
Delivering endocrinology and reproduction in an integrated modular curriculum (Ghayur et al., 2012)	Medical students at Shifa College of Medicine, Pakistan. (*N* = 86)	Integrated modular curriculum (spiral). **Learning methods**: LGID, SGD, SDL, Role Plays and Integrated Practical Sessions.	**Number of questions**: 7 **Scale**: 3-point Likert scale (Agree, Neutral, Disagree)	NR
	Faculty members involved in the delivery and assessment of the integrated module (*N* = 14).		**Number of questions**: 5 **Scale**: 5-point Likert scale (Strongly agree, Agree, Neutral, Disagree, Strongly Disagree)	NR
Developing tool and measuring integration characteristics of basic science curriculum to improve curriculum integration (Maharjan, 2018)	Faculty who developed the basic science curriculum (*N* = 20) Medical students (*N* = 525)	Integrated basic science curriculum **Learning methods**: PBL	**Number of questions**: 20 **Scale**: 4-point Likert scale (1: Strongly disagree, 2: Disagree, 3: Agree and 4: Strongly agree)	**Reliability**: ▪ Internal consistency: Highly reliable with Cronbach’s Alpha: 0.883 ▪ Consensus analysis for the 20 characteristics lies between 77.31–85.45%
Survey of pharmacy schools’ approaches and attitudes toward curricular integration (Poirier T. et al., 2016)	Academic leaders (*n* = 376) representing 94 pharmacy schools.	NR	**Number of questions**: 15 ▪ 11 MCQs ▪ 3 questions: 6-point Likert scale (Strongly agree, Agree, neither agree nor disagree, Disagree, Strongly disagree, No opinion) ▪ One open ended question	NR
Integration of basic and clinical science courses in US PharmD programs (Islam M. et al., 2016)	Faculty members from accredited PharmD programs (*N* = 126)	NR	**Number of questions**: 25 **Scale**: 5-point Likert scale ranging from 1: strongly disagree to 5: strongly agree. (sections 1 and 2)	**Face validity**: Two independent pharmacy faculty members reviewed the survey.
Evaluation of the first curriculum year of the new integrated and interactive curriculum at the university of medicine and pharmacy at Ho Chi Minh city (ump), Vietnam (Le B. K., 2018)	First year medical students (*N* = 393)	Interactive and integrative. **Learning methods**: TBL	**Number of questions**: 30 **Scale**: 4-point Likert scale: (strongly disagree, disagree, agree, strongly agree)	NR
	Faculty members (*N* = 48)		**Number of questions**: 40 **Scale**: 4-point Likert scale: (strongly disagree, disagree, agree, strongly agree)	NR
Evaluating dental students’ perspectives on the concurrent teaching of didactic and case-based courses (Parikh et al., 2022)	Dental students (years 1–4) (*N* = 229)	Integrated learning model **Learning methods**: Didactic and CBL	**Number of questions**: 7 **Scales**: 5 points Likert-scale; 1: “Strongly Disagree” to 5: “Strongly Agree”. 2 open-ended questions regarding what students enjoyed in each course and suggestions for improvement.	NR
An integrated learning curriculum for radiography in South Africa (Engel-Hills P. C., 2005)	Radiographers Radiographer students and lecturers.	ILC **Learning method**: SDL, SGD (≤ 10 students).	*Section G* (program design): **Number of questions**: 24 items **Scales**: 4-points Likert scale (Always, mostly, sometimes, never).	NR
Evaluating the construct validity of basic science curriculum assessment instrument for critical thinking: a case-study (Chen C. et al., 2018)	Medical students (Years 1 and 2) Medical graduates	Hybrid integrated curriculum	**Number of questions**: 22 **Scale**: 5-point Likert scale (0: “Not Applicable”, 1: “Strongly Disagree”, 2: “Disagree”, 3: “Agree”, and 4: “Strongly Agree”).	**Reliability**: The Cronbach-α: 0.97 (high) **Uni-dimensionality**: Eigenvalue: 3.08 (violation of uni-dimensionality). **Content validity**: ▪ Review of critical thinking literature and Association of American Medical College (AAMC) graduating student surveys. ▪ The survey was validated by an academic dean of the medical school (expert).
Evaluation of Integrated Teaching Method for Phase I MBBS, Using Kirkpatrick’s Evaluation Method (Dohnde et al., 2020)	First year MBBS students	Integrated curriculum **Learning method**: Didactic lectures, case scenarios.	**Number of questions**: 26 **Scale**: 5-point Likert scale: (1 - Poor, 2 - Satisfactory, 3 - Good, 4 - Better And 5 - Excellent.)	NR
	Faculty members		**Number of questions**: 10 **Scale**: 5-point Likert scale: (1 - Poor, 2 - Satisfactory, 3 - Good, 4 - Better And 5 - Excellent.)	NR
Simulated patient videos to supplement integrated teaching in competency based undergraduate medical curriculum (Nayak K. R. et al., 2023)	First year MBBS students at Kasturba Medical college Manipal. Faculty members.	Competency-based medical curriculum **Learning methods**: Clinical cases	**Number of questions**: 24 questions with 5-point Likert scale, 3 preference questions (choosing the preferred educational method) and two open ended questions. **Scale**: 5- Likert scale (1 and 2: Agree, 3: Neutral, 4 and 5: Disagree)	**Reliability**: ▪ Internal consistency: Cronbach’s alpha = 0.97 (high) ▪ ICC > 0.30 (for all items); high discriminant capacity. ▪ PCA, KMO = 0.934 **Content and face validity**: ▪ Experts’ opinion on the questionnaire was obtained.
An integrated curriculum: evolution, evaluation, and future direction (Howard K. et al., 2009)	Course directors (*N* = 38) representing 84 courses.	NR	**Response options**: From this list of 10 levels of integration model select the model which illustrate your course framework and design: ▪ Single discipline (fragmented, connected, nested) ▪ Multiple discipline (sequenced, shared, threaded, webbed, integrated) ▪ Learner model: (networked, immersed).	NR
A tool for evaluating session‑level integration in medical education (Heck A., Chase A., 2021)	Health science educators and faculty members.	NR	**Number of questions**: 12 **Scale**: Rating ranges from 1–4 in each domain. **Session integration profile score =** Design + Delivery + outcomes. **Integration profile score interpretation**: 3–4: Isolation 5–7: Initial 8–10: Intermediate 11–12: Mature	**Content and face validity**: Literature review and tool evaluation by medical educators. **Construct validity**: Applying the tool to sample case studies **Inter-rater reliability**: ▪ Interclass correlation coefficient: ▪ Intermediate case study: 0.97 (95% CI 0.88–0.99) ▪ Isolated case study: 0.96 (95% CI 0.88–0.99) **Factor analysis (number of domains)**: One factor (integration) was found and it is composed on the 3 items explaining 46.156% of the variance (factor ladings 0.491–0.764).
Level of integration in current undergraduate curricula of two private-sector medical colleges in Karachi (Baig et al., 2022)	Basic science and clinical instructors at two private medical colleges in Karachi, Pakistan.	Integrated curriculum (spiral approach) **Learning methods**: PBL	**Number of questions**: ▪ 11 close-ended questions (steps of Harden’s integration ladder) ▪ 6 open-ended questions (perspectives on current integration strategies) **Scale**: 5-point Likert scale: for questions 3–11 (1: never, 2: rarely, 3: sometimes, 4: mostly, 5: always) Questions 1 and 2 are reverse scored. *Integration score*: Adding scores per response to one of the five options. Total integration score is 55. **Completion time**: 15–25 min	**Face and content validity**: Expert opinions. **Reliability**: *Internal consistency*: Cronbach’s alpha:0.732 *Principal Component Analysis (PCA)*: ▪ KMO = 0.733, Bartlett’s test *P* < 0.001, *r* ≤ 0.5 ▪ Three component model ▪ Eigen value > 1, cumulative variance explained = 57.1%, questions loading (0.5–0.9).

In terms of the instruments’ content, the number of questions varied widely, with the lowest reported being 4 in the *Integrated standardized patient examination assessment rubric* [37], while the largest set of questions was found in the *Integrated curriculum in nursing inventory* consisting of 138 questions divided into 3 different Sect [26]. *The integrated curriculum in nursing inventory* is a comprehensive questionnaire that evaluates the participant understanding of curriculum integration, their perception of the current integrated curriculum, and their views on how an integrated curriculum should be. The response options for all close-ended questions in the identified instruments within this review were presented using a Likert scale. Notably, the 5-point Likert scale was the most commonly employed scale in this systematic review.

Our findings suggest that the validation of instruments’ assessing integration was uncommon. Of the 22 instruments identified in this review, only 9 underwent assessment for psychometric properties. The most frequently reported psychometric property among the included instruments was content validity (*n* = 7). The instruments analyzed in this study can be classified into three main groups based on their objectives:

- Instruments assessing **integration attributes** through students’ performance (outcome of curriculum integration).
- Instruments assessing participants’ (students or faculty members) **perception about the integrated curriculum characteristics, process, and outcomes**.
- Instruments evaluating curriculum **integration level based on participants’ experiences** (e.g. reviewing integration introduction in the health professions’ curricula of a country, assessing the level of curriculum integration in an institution).

### Instruments assessing integration attributes

Three instruments assessing students’ ability to integrate knowledge were identified in this review. These instruments were developed by Panzarella (2003) to assess integration attributes of physical therapy students during an OSCE exam [26]. One of these instruments is *the Integrated Standardized Patient Examination* (ISPE), which functions as an assessment rubric for integration. It evaluates students’ competency in integration by assessing their responses to integration-related questions posed by standardized patients. The remaining two instruments were questionnaires designed to investigate perceptions of students’ performance, specifically regarding integration competencies, during the OSCE interaction with the standardized patients (SP). These questionnaires were intended for both students and expert evaluators. The ISPE was validated for validity and reliability measures. A definition of integration was provided in the beginning of the feedback assessing instruments. Detailed findings are reported in Table 1.

### Instruments assessing perceptions about integrated curriculum characteristics, process, and outcomes

Instruments in this category evaluate integrated curricula’s characteristics, including aspects of delivery and implementation. Several of these instruments also explore participants’ perceptions about the usefulness of the curriculum in terms of achieving desired outcomes of curriculum integration. The majority of questionnaires identified in this review fall into this category (*n* = 16) [28–38, 40]. Many of these instruments explore participants’ opinions on different aspects of the integrated curriculum, such as the content, delivery, time-management, and teaching methods. Examples of these instruments include *The Questionnaire Assessing Students’ Perception Regarding an Integrated Curriculum at a Public Sector Medical Colleg*e, *The Integration Characteristic Tool* and *The Basic Science Curriculum Assessment Instrument* [28, 30, 34]. Some instruments like *The Integrated Curriculum Implementation Inventory* and *Student and Faculty Online Survey Questionnaires developed* by Le BK [31]. assess the degree of integrated curriculum implementation. One unique instrument within this category is *The Integrated Curriculum in Nursing Inventory*, which assesses the respondent’s view and understanding of the “integrated curriculum” concept at the beginning of the questionnaire before evaluating their perception of the current curriculum [37]. Additionally, this is the only instrument which enables the participants to express their perceptions of the required changes for an ideal integrated curriculum.

Two of the identified instruments; *The integration survey* [38] and *Faculty perception of curricular integration survey* [40] were developed to assess curricular integration in pharmacy programs on a national level. Therefore, the target sample for these instruments are faculty members involved in the delivery of integrated curricula and academic leaders from institutions adopting integrated curricula. Many of the studies cited in this review assessed curriculum integration through the perceptions of students, faculty members and academic leaders.

### Instruments evaluating curriculum integration levels

This category includes three instruments, *The Integrated Curriculum Evaluation Instrument* by Howard et al. (2009), the *Integration Ladder Questionnaire*, and *The Session Integration Tool (SIT)* [27, 39, 41]. These three instruments are the most specific tools among all the integration instruments identified as they assess the degree of curriculum integration and provide a detailed description of the curriculum integration level. The first two of these instruments (*The Integrated Curriculum Evaluation Instrument* by Howard et al., *Integration Ladder Questionnaire*) were developed based on established integration models; the Fogarty models for curriculum integration [11] and Harden’s integration ladder [10] respectively.

Unlike the other two instruments in this category, the *SIT* has a narrower scope as it assesses the degree of integration within one session and not across the whole curriculum [27]; however, it has been adequately validated and has a clear score calculation approach. The scores are then interpreted into one of four categories each representing a level of integration. Likewise, *The integration ladder questionnaire* [39] has a reported method for integration score calculation, the mean score is then used to determine the integration level on Harden’s ladder. Further details about the instruments and their psychometric properties are described in Table 2.

The majority of the studies included in the review were found to have low risk of bias with 2 instruments scoring 7 while many of them scoring between 5 and 6 out of the 7 overall score (*n* = 9). On the other hand, five of the papers had score of 4 or less. Details of the quality assessment results are reported in Table 3.

**Table 3:** Quality assessment of study articles based on the Risk of Bias Utilized for Survey Tool (ROBUST)

Study (author, year)	Sampling frame	Participant recruitment	Sample size	Sample characteristics	Measurement validity	Setting	Data management	Overall score
Panzarella, K. 2003	0	0	1	1	0	1	1	4
Lajber et al., 2020	1	0	1	1	0	1	0	4
Strandell, CH., 1980	1	1	1	1	1	1	0	6
Ghayur et al., 2012	1	0	1	0	0	1	0	3
Maharjan, 2018	1	1	1	0	1	1	0	5
Poirier T. et al., 2016	1	1	1	1	0	1	0	5
Islam M. et al., 2016	1	1	1	1	0	1	1	6
Le B. K., 2018	1	1	1	1	0	1	0	5
Parikh et al., 2022	1	1	1	1	0	1	1	6
Engel-Hills P. C., 2005	1	1	1	0	1	1	0	5
Chen C. et al., 2018	0	1	1	0	1	0	0	3
Dohnde et al., 2020	1	1	1	1	0	1	0	5
Nayak K. R. et al., 2023	1	1	1	1	1	1	1	7
Howard K. et al., 2009	1	1	1	1	1	1	1	7
Heck A., Chase A., 2021	1	1	1	0	0	1	0	4
Baig et al., 2022	1	1	1	1	1	1	0	6

## Discussion

This systematic review successfully addresses the research question by identifying articles that report on instruments evaluating the degree of curriculum integration in health professions’ education. This review identified twenty-two instruments focused on evaluating the degree of curriculum integration in health professions’ curricula. Curriculum evaluation is a process which focuses on obtaining information about different components of the curriculum [42]. There are various sources for information regarding the curriculum; however, the majority of instruments identified in our review focus on obtaining the input of students and faculty members who are the end users of a health professions curriculum.

The studies reviewed in this analysis examined the curricula of various health professions, with a predominant emphasis on medical education programs. This outcome was anticipated, as the concept of “curriculum integration” has traditionally been closely associated with medical education. However, it’s noteworthy that the concept’s application has gradually extended beyond medical training to encompass other fields within healthcare education [1]. The instruments were classified based on the objective of their assessment into three categories; instruments assessing integration attributes, instruments assessing perceptions about integrated curriculum characteristics, processes and outcomes, and instruments assessing integration level.

The first category focuses on instruments evaluating students’ integration competency within the context of an OSCE assessment. *ISPE*, is a rubric that assesses integration competency through students’ responses to integration specific questions asked by the SP [26]. Integration competency fulfillment represents the ultimate objective of an integrated curriculum. Here, integration occurs within the student’s mind as they synthesize all acquired knowledge and skills from across the curriculum. The comprehensive understanding enables the students to effectively apply the integrated knowledge in making informed decisions during clinical practice. This also represents the highest level of integration in both Harden’s and Fogarty’s integration models; known as the transdisciplinary level (or fusion) and the immersed model, respectively [10, 11]. The ISPE rubric stands out as a highly effective tool for evaluating curriculum integration due to its unique focus on assessing the outcome of integration. By objectively examining students’ integrative capacity and their proficiency in applying knowledge and skills across diverse contexts within the curriculum, including real-world scenarios, the ISPE rubric offers a comprehensive evaluation framework. Its emphasis on evaluating not just the process of integration, but also its tangible impact on students’ abilities to navigate real-world challenges, underscores the robustness and relevance of the ISPE rubric in educational assessment. This makes it a valuable resource for educators measuring the effectiveness of curriculum integration efforts and the practical readiness of students for professional practice [26]. 

Intriguingly, student performance in the ISPE was also evaluated using questionnaires designed to solicit feedback from students and other observers who have witnessed the interaction. Considering the importance of reflection as an effective strategy for enriching the learning experience in complex subjects and fostering a deeper understanding of professional values [43]. The student feedback questionnaire is an excellent instrument which provides students with opportunity to reflect on their performance and determine areas requiring improvement.

The second category of the integration assessing instruments is the largest; containing 16 instruments developed for students, faculty members, and other academic staff. These instruments explore participants’ views on the integrated curriculum, its characteristics, implementation, and outcomes. The characteristics of integrated curricula assessed were related to the content of the curriculum, delivery, teaching methods, and student assessment (*n* = 9). An important component of curriculum integration, which was evaluated by many of the identified instruments, was content coherence. Content coherence is a necessary pre-requisite for realizing full curriculum integration as the whole curriculum cannot be correlated and made more meaningful to the learner if individual components are not coherent [42]. Integration in education involves the seamless blending of existing knowledge with new learning, creating a cohesive and interconnected educational experience. This process emphasizes the importance of organizing curriculum content in a coherent manner, where different subjects, concepts, and skills are coordinated and presented in a unified framework. By aligning learning objectives, topics, and activities across various disciplines and modules, educators can facilitate a more holistic understanding of the subject matter. Content coherence ensures that students can recognize connections between different topics and apply their learning in diverse contexts, promoting deeper comprehension and more effective transfer of knowledge and skills. Additionally, it fosters a sense of coherence and continuity in the curriculum, enhancing the overall learning experience for students. The tools mentioned above can thus serve as a crucial framework for curriculum development and as a standard for improving curricular quality. Many of the instruments extracted in this study explored opinions on integrated curriculum delivery including teaching and learning methods, preparation for class, and interactions between students and faculty within class. These elements are important as they impact the extent to which integration is achieved within individual sessions and in the curriculum as a whole. Some of the items also assessed faculty development activities which prepare them to contribute to curriculum integration. Faculty members’ preparation for their role within an integrated program is crucial because the success of integration is impacted by the instructors’ understanding of their role, the role of others, and how they can coordinate with other faculty members to help students understand the link between different subjects and disciplines [42, 44].

Two of the identified instruments; *the integration characteristic tool* [30] and *the Basic Science Curriculum Assessment Instrument* [34] only measure horizontal integration as they were developed to assess perceptions regarding integration in basic science curricula. These instruments could be used during initial stages of integration for institutions introducing horizontal integration within the basic science discipline. A study published by Brynhildsen J et al. (2002) has shown that both students and faculty members view horizontal and vertical integration as important components of medical curricula, with a general belief that horizontal integration might be more important [45]. Revisiting foundational knowledge from basic sciences during clinical courses and practical experiences results in deeper understanding of basic knowledge, especially when it is linked with real life applications.

The implementation of integrated curricula was assessed in two of the instruments; *The Integrated Curriculum Implementation Inventory* by Strandell C. *(1980)* [37], and the *Students’ and Faculty’s Online Survey Questionnaires* developed by Le B. (2018) [31]. The aforementioned tools included meticulously crafted questions assessing how integration was implemented before and during class, as well as during lectures and student assessments. This is very useful in providing valuable insights into the effectiveness and thoroughness of integration efforts within the curriculum. Additionally, these instruments also evaluated the preparedness of faculty members to facilitate curriculum integration, a crucial aspect in integrated medical curricula [44]. .

The last category of the instruments identified by this review evaluate the degree or level of curriculum integration. Two of these instruments; *The Integrated Curriculum Evaluation Instrument* by Howard et al. (2009) [41] and the *Integration Ladder Questionnaire* [39] were developed based on Fogarty’s model for curriculum integration and Harden’s integration ladder, respectively [10, 11]. While Howard’s *Integrated curriculum evaluation instrument* assesses the extent of vertical and horizontal integration, its main limitation is that it provides a qualitative assessment of the curriculum which cannot be standardized and might be more susceptible to bias, and its use is restricted to course directors [41]. *The integration Ladder Questionnaire* [39] is a user-friendly, quantitative instrument whose target audience are educators and faculty members. This questionnaire consists of 11 close-ended questions, each of which represents a step on the integration ladder. The lowest step of the ladder represents a subject-based curriculum, with increased integration as you move you up the ladder. This instrument has been ratified for validity and reliability measures, and was found to have adequate internal consistency. The *SIT* is the last instrument in this category, which assesses integration within a single session. This is a quantitative instrument which has a simple and clear criterion to assess the degree of curriculum integration within a session [27]. This instrument was assessed for content and construct validity, inter-rater reliability, and factor analysis.

Our systematic review reveals that the topic of integration assessing instruments’ development is an active research area, which has become popular with increased adoption of integrated educational models. More than half of the studies included in this review were published after 2010. Although many instruments have been identified in the literature, only a few were validated to assess their psychometric properties. This is a limitation for the use of these instruments for future curriculum evaluation since their reliability and validity is still unknown. Therefore, this is an area which needs to be explored and studied further in future.

Notably, the majority of the identified assessment tools are limited in their ability to measure the degree of curriculum integration. Rather than providing quantitative evaluations of integration levels, these tools primarily focused on gathering qualitative data in the form of perceptions and feedback from stakeholders. This sheds the light on a substantial gap in the field of integrative medical curriculum assessment. The identified shortcoming necessitates the development of more robust evaluation methods capable of quantitatively assessing the degree of integration within medical curricula. Addressing this gap is essential for ensuring that educational programs effectively meet the goals of integration and adequately prepare students for the complex challenges of contemporary healthcare practice. To our knowledge, this systematic review is the first study in the literature to identify and describe the characteristics of tools assessing the degree of curriculum integration. Our search was comprehensive and included 8 major academic databases, in addition to further manual searching, although there is a possibility that relevant studies published in languages other than English might have been missed. In addition, qualitative instruments that assess curriculum integration were not included as our review focused on identifying quantitative instruments. Our inclusion criteria were very specific, excluding instruments not assessing the degree of curriculum integration such as those focused on assessing PBL [46–49]. Our study revealed the scarcity of validated instruments assessing curriculum integration in the literature, which highlights the need for more validation studies on currently available instruments.

## Conclusion

Curriculum integration is a contemporary concept in the field of medical education which has been widely adopted by different health professions schools globally to optimize the students’ educational experience and prepare them for practice. To assess the extent to which integration has been fulfilled within these curricula, different instruments were developed. Our study aimed to identify these instruments and extract their psychometric properties. The results of this systematic review report on numerous instruments designed to assess the extent of curriculum integration within health professions’ educational programs. The majority of these instruments explore participants’ perceptions of the characteristics of the integrated curriculum including assessment of curricular content, delivery, and implementation. This study also identified tools which provide a broad approach for integration score calculation and determination of integration level. It is important to note that the majority of these instruments have not been validated and therefore further assessment of their psychometric properties is required. Furthermore, there is a necessity to create instruments that are both sensitive and specific, and are tailored to accurately gauge the level of curriculum integration within medical curriculum.

### Electronic supplementary material

Below is the link to the electronic supplementary material.

Supplementary Material 1

## Data Availability

Data is provided within the manuscript or supplementary information files.

## Abbreviations

CBL: Case Based Learning
ILC: Integrated Learning Curriculum
ISPE: The Integrated Standardized Patient Examination
LGID: Large Group Interactive Sessions
MBBS: Bachelor of Medicine, Bachelor of Surger
MCQs: Multiple Choice Questions
NR: Not Reported
OSCE: Objective Structured Clinical Examination
PBL: Problem based Learning
PRISMA: The Preferred Reporting Items for Systematic reviews and Meta-Analyses
SDL: Self- directed Learning
SGD: Small Group Discussions
SIT: Session Integration Tool
SPICES: Student-centered, Problem-based, Integrated, Community-based, Elective, Systematic
TBL: Team Based Learning
