# Supplementary material for: Tools for measuring curriculum integration in health professions’ education: a systematic review
Source: BMC Med Educ. 2024 Jun 6;24:635. doi: 10.1186/s12909-024-05618-5 (PMC11157845; doi:10.1186/s12909-024-05618-5)
Supplement: Supplementary file 1 — Supplementary Material 1 [file 12909_2024_5618_MOESM1_ESM.docx]

**Additional file 1 (.docx)**. **PubMed search strategy**

**Research question:**

- What tools or instruments or surveys are available (developed and published by researchers in peer reviewed journals or other academic platforms) for measuring the degree of integration in a PBL based curriculum in health professions schools?

**Search categories:**

- Tool, instrument, survey, questionnaire, scale, measure
- (Curriculum delivery)) OR (curriculum evaluation)) OR (curriculum assessment)
- integrated curriculum, vertical integration, horizontal integration, spiral integration, basic sciences integration, clinical sciences integration, clinical and basic sciences’ integration,
- Medical education, medical school, medical college, health professions education
- Problem based learning, PBL, Student centered curriculum

**Search strategy:**

(((((((((Tool) OR (instrument)) OR (questionnaire)) OR (survey)) OR (scale)) OR (measure)) AND (((Curriculum evaluation) OR (Curriculum assessment)) OR (curriculum delivery))) AND (((((((Integrated curriculum) OR (vertical integration)) OR (horizontal integration)) OR (spiral integration)) OR (basic sciences integration)) OR (clinical sciences integration)) OR (clinical and basic sciences' integration))) AND ((((Medical education) OR (Medical school)) OR (Medical College)) OR (health professions education))) AND (((Problem based learning) OR (PBL)) OR (student centered learning))

2/10/2023 >>>> Results: 683
